# Advantages and detection of phase coding in the absence of rhythmicity

**DOI:** 10.1002/hipo.23199

**Published:** 2020-02-17

**Authors:** Daniel Bush, Neil Burgess

**Affiliations:** ^1^ UCL Institute of Cognitive Neuroscience London UK; ^2^ UCL Queen Square Institute of Neurology London UK

**Keywords:** entorhinal cortex, grid cells, hippocampus, neural coding, oscillations, place cells

## Abstract

The encoding of information in spike phase relative to local field potential (LFP) oscillations offers several theoretical advantages over equivalent firing rate codes. One notable example is provided by place and grid cells in the rodent hippocampal formation, which exhibit phase precession—firing at progressively earlier phases of the 6–12 Hz movement‐related theta rhythm as their spatial firing fields are traversed. It is often assumed that such phase coding relies on a high amplitude baseline oscillation with relatively constant frequency. However, sustained oscillations with fixed frequency are generally absent in LFP and spike train recordings from the human brain. Hence, we examine phase coding relative to LFP signals with broadband low‐frequency (2–20 Hz) power but without regular rhythmicity. We simulate a population of grid cells that exhibit phase precession against a baseline oscillation recorded from depth electrodes in human hippocampus. We show that this allows grid cell firing patterns to multiplex information about location, running speed and movement direction, alongside an arbitrary fourth variable encoded in LFP frequency. This is of particular importance given recent demonstrations that movement direction, which is essential for path integration, cannot be recovered from head direction cell firing rates. In addition, we investigate how firing phase might reduce errors in decoded location, including those arising from differences in firing rate across grid fields. Finally, we describe analytical methods that can identify phase coding in the absence of high amplitude LFP oscillations with approximately constant frequency, as in single unit recordings from the human brain and consistent with recent data from the flying bat. We note that these methods could also be used to detect phase coding outside of the spatial domain, and that multi‐unit activity can substitute for the LFP signal. In summary, we demonstrate that the computational advantages offered by phase coding are not contingent on, and can be detected without, regular rhythmicity in neural activity.

## 1 INTRODUCTION

In the central nervous system, phase coding refers to the encoding of information in the phase of neuronal activity with respect to an ongoing oscillation in the local field potential (LFP). Theoretically, the coding of information in spike phase offers numerous advantages over a pure rate code. Phase coding can be used to facilitate rapid pattern classification (Thorpe, Delorme, & Van Rullen, 2001) and incurs a lower metabolic cost than an equivalent spike rate code (Fries, Nikolic, & Singer, 2007). It may also be useful for the multiplexing of information (Panzeri, Brunel, Logothetis, & Kayser, 2010); disambiguating stimuli that generate similar spike rates (Kayser, Montemurro, Logothetis, & Panzeri, 2009; Montemurro, Rasch, Murayama, Logothetis, & Panzeri, 2008); and preventing interference between simultaneously presented stimuli (Fries et al., 2007; Jensen et al., 2014). Empirically, phase coding has been identified across multiple species and cortical regions (see Fries et al., 2007; O'Keefe & Burgess, 2005; Panzeri et al., 2010 for reviews). For example, information encoded in spike phase exceeds that encoded in spike rate alone in the primate visual (Montemurro et al., 2008), prefrontal (Siegel, Warden, & Miller, 2009), and auditory (Kayser et al., 2009) cortices, as well as in rodent somatosensory cortex (Zuo et al., 2015); while firing phase in the macaque superior temporal sulcus has been shown to differentiate visual stimulus categories (Turesson, Logothetis, & Hoffman, 2012). However, the best known example of phase coding comes from place and grid cells in the rodent hippocampal formation, whose firing phase relative to the ongoing 6–12 Hz movement related theta oscillation advances progressively as their spatial firing fields are traversed (Hafting, Fyhn, Bonnevie, Moser, & Moser, 2008; O'Keefe & Recce, 1993). Importantly, this theta phase precession is independent of both running speed (Geisler et al., 2010; Geisler, Robbe, Zugaro, Sirota, & Buzsáki, 2007) and heading direction (Climer, Newman, & Hasselmo, 2013; Huxter, Burgess, & O'Keefe, 2003; Jeewajee et al., 2014), ensuring that firing phase provides more spatial information than spike rate alone (Jensen & Lisman, 2000).

To date, it has often been assumed that the absence of a baseline oscillation with relatively constant frequency and high amplitude, characterized by a narrow peak in the LFP power spectrum or spike train temporal auto‐correlogram, is evidence for a lack of phase coding (Yartsev, Witter, & Ulanovsky, 2011; but see Barry, Bush, O'Keefe, & Burgess, 2012). Nonetheless, firing phase is independent of frequency, such that phase coding can be implemented with a baseline oscillation that varies dynamically over a wide range of frequencies (Blair, Wu, & Cong, 2013; Orchard, 2015). Indeed, it has recently been demonstrated that place cells in bat hippocampus exhibit both phase locking and phase precession relative to aperiodic, low frequency fluctuations in the LFP (Bush & Burgess, 2019; Eliav et al., 2018). Experimental evidence also hints at such a scheme in the human brain, where narrow peaks in power spectra are rare but spike‐triggered LFP averages indicate that neural firing is phase locked to ongoing oscillations (Jacobs et al., 2013; Jacobs, Kahana, Ekstrom, & Fried, 2007). This is supported by behavioral evidence showing that increased spike phase coherence correlates with mnemonic performance, even in the absence of an associated peak in the LFP power spectrum (Rutishauser, Ross, Mamelak, & Schuman, 2010).

Here, we demonstrate that spike phase can robustly encode information in the absence of a constant frequency, high amplitude baseline oscillation. As an example, we examine the phase code for location exhibited by grid cells of the medial entorhinal cortex. Using LFP recordings from depth electrodes in the hippocampal formation of pre‐surgical epilepsy patients performing a spatial memory task (Bush et al., 2017), we demonstrate that phase precession in simulated grid cells can be robustly maintained in the absence of any clear peak in the LFP power spectra or spike train temporal auto‐correlogram, consistent with recent data from flying bats (Eliav et al., 2018). We then demonstrate that this allows simulated grid cells to multiplex information beyond that encoded in firing rate alone (Fiete, Burak, & Brookings, 2008; Mathis, Herz, & Stemmler, 2012), with firing phase indicating movement direction analogous to experimentally observed “theta sequences” of activity along an animal's current trajectory (Burgess, Recce, & O'Keefe, 1994; Johnson & Redish, 2007; Skaggs, McNaughton, Wilson, & Barnes, 1996; Zutshi, Leutgeb, & Leutgeb, 2017). Extending previous observations in place cells (Jensen & Lisman, 2000), we also show that firing phase can be used to improve the accuracy of decoding location from firing rates alone, which can suffer when grid cells exhibit stable differences in in‐field firing rate (Boccara, Nardin, Stella, O'Neill, & Csicsvari, 2019; Butler, Hardcastle, & Giocomo, 2019; Ismakov, Barak, Jeffery, & Derdikman, 2017). Finally, we describe analytic techniques that can be used to identify phase coding in the absence of high amplitude and continuous frequency oscillation. In particular, we show that dividing the receptive field or spike train into a small number of discrete regions and then extracting the mean phase in each region after filtering the LFP signal in a broad frequency range allows the robust identification of phase coding. Indeed, a similar approach has recently been used to identify grid and place cell phase precession against highly variable low frequency oscillations in the flying bat (Eliav et al., 2018). In summary, we demonstrate that phase coding in grid cells is not contingent on sustained rhythmicity in neural activity and offers several computational advantages over a pure rate code.

## 2 METHODS

### 2.1 Grid cell rate code

We simulate a total of *N* = 200 grid cells divided equally among *m* = 5 modules with a minimum scale of *s*_5_= 30 cm and a ratio of 1.4 between subsequent scales *s*_*m*_. We make use of a phenomenological model of grid cell firing in which the relative influence of rate and phase coding can be independently modulated (following Chadwick, van Rossum, & Nolan, 2015). Specifically, the firing rate of each cell *r*_*g*_(*t*) at time *t* is the product of a rate code$\;r_{x}(\vec{x}$) that is dictated by the agent's location$\;\vec{x}\left(t\right)=\left\{x,y\right\}$; and a phase code *r*_*ϕ*_(*ϕ*, *θ*) that is dictated by the location‐dependent preferred firing phase $\phi \left(\vec{x}\right)$ and LFP phase *θ*(*t*):

(1) $$r_{g}\left(t\right)=r_{x}\left(\vec{x}\left(t\right)\right)\;r_{\phi }\left(\phi \left(\vec{x}\left(t\right)\right),\theta \left(t\right)\right).$$

The grid cell rate code$\;r_{x}(\vec{x}$) is a Gaussian function of the distance *d* between the agent and the center of the closest grid node$\;\vec{x_{c}}=\left\{x_{c},y_{c}\right\}$, with field width governed by a constant *σ*_*m*_ = *s*_*m*_/10 and the maximum in‐field firing rate given by a constant *r*_*c*_:

(2) $$r_{x}\left(\vec{x}\right)=r_{c}\;\exp\left(\frac{-d^{2}}{2{\sigma_{m}}^{2}}\right).$$

In simulations of movement on a linear track, the peak locations of each grid firing field $\vec{x_{c}}$ are uniformly distributed and repeat with a period equivalent to the grid scale. In simulations of movement in a two‐dimensional (2D) environment, the peak locations of each grid firing field $\vec{x_{c}}$ are uniformly distributed and repeat at the vertices of a rhombus with the length of each side equal to the grid scale and an acute angle of 60°.

In simulations of a uniform grid firing pattern, the value of maximum in‐field firing rates *r*_*c*_= 1; while in simulations where firing rates vary between grid fields (as observed experimentally; Ismakov et al., 2017), the specific value for each field is drawn at random from a normal distribution with a mean and variance of one (rectified to prevent negative firing rates).

### 2.2 Grid cell phase code

The grid cell phase code *r*_*ϕ*_(*ϕ*, *θ*) is modelled as a circular Gaussian function of the difference between instantaneous LFP phase *θ*(*t*) and the location‐dependent preferred firing phase of each cell $\phi \left(\vec{x}\right)$, with the influence of phase coding over the spike train governed by a constant *k*= 1.5:

(3) $$r_{\phi }\left(\phi ,\theta \right)=\exp\left(k\cos\left(\phi \left(\vec{x}\right)-\theta \left(t\right)\right)\right).$$

In simulations of grid cells that exhibit phase precession, the location dependent preferred firing phase of each cell $\phi \left(\vec{x}\right)$ is proportional to the linear distance from the current location to the centre of the closest grid node projected onto the direction of travel$\;d_{\phi }=\hat{v}∙\vec{d}$, where $\hat{v}$ is a unit vector in the direction of velocity $\vec{v}$ (following Burgess et al., 1994; Jeewajee, Barry, O'Keefe, & Burgess, 2008a):

(4) $$\phi \left(\vec{x}\right)=2\pi \left(\frac{d_{\phi }}{s_{m}}+0.5\right).$$

Conversely, in simulations of grid cells that exhibit phase locking, the preferred firing phase *ϕ* = *π* at all locations within the environment.

The overall activity of each grid cell *R*(*t*) is determined by the product of the firing rate *r*_*g*_(*t*); the instantaneous baseline frequency *f*(*t*), to normalize the number of spikes in each oscillatory cycle; and instantaneous running speed $\left\|\vec{v}\right\|$ multiplied by a constant *m*_*v*_ = 0.16 cm^−1^, to account for the experimentally observed increase in firing rate with running speed (Sargolini et al., 2006):

(5) $$R\left(t\right)=r_{g}\left(t\right)f\left(t\right)m_{v}v\left(t\right).$$

Finally, spike trains for each grid cell are generated by an inhomogeneous Poisson process with rate *r*_tot_(*t*), which is determined by *R*(*t*) and normalized to ensure that each cell has a mean firing rate of $\bar{r}=$ 2 Hz across the duration of the simulation *T*:

(6) $$r_{\mathrm{tot}}\left(t\right)=\frac{\bar{r}\mathrm{TR}\left(t\right)}{\int_{0}^{T}R\left(t\right)\mathrm{dt}}.$$

### 2.3 Movement trajectories

Behavioral trajectories in one‐dimensional (1D) correspond to 300 s movement along a linear track with a sample rate of 200 Hz and running speed *v*(*t*) that varies randomly over time in the 2–30 cm s^−1^ range. Running speed is integrated over time to compute linear displacement along the track *x*(*t*), with an average total track length of ~50 m. Behavioral trajectories in 2D are taken from recordings of rats running for scattered food rewards in a 1 m sided square environment, sampled at 50 Hz (Barry, Hayman, Burgess, & Jeffery, 2007). These are up‐sampled by linear interpolation to provide location coordinates *x*(*t*) and *y*(*t*) at a sample rate of 200 Hz, from which running speed $\left\|\vec{v}\right\|$ is computed as the linear displacement in each time step.

### 2.4 LFP signal

In initial simulations that aim to replicate the properties of rodent entorhinal grid cells, the LFP signal is a sinusoid with frequency *f*= 8 Hz. In further simulations that aim to examine the properties of putative human grid cells, the LFP signal is taken from depth electrodes in the hippocampi of pre‐surgical epilepsy patients performing a spatial memory task at the National Hospital for Neurology and Neurosurgery, London, recorded at a sample rate of 512 Hz (see Bush et al., 2017 for further details). In this case, we first examine post‐implantation computed tomography scans co‐registered with pre‐implantation magnetic resonance images to identify candidate electrode contacts. We choose those which are unequivocally located in the body of the hippocampus, as the fidelity of post‐implantation images is not sufficient to confidently resolve medial entorhinal cortex. However, rodent data suggest that movement related oscillatory activity is highly coherent between these regions (Mizuseki, Sirota, Pastalkova, & Buzsaki, 2009).

Next, the LFP signal from hippocampal electrode contacts is band‐pass filtered in the 2–20 Hz range using a zero‐phase, second‐order Butterworth filter. The specific choice of filter band is arbitrary, as any LFP signal with broadband low frequency power from which the phases of multiple spikes can be extracted after filtering is sufficient (i.e., giving an upper limit of several hundred Hz, assuming spikes have duration of ~1 ms). Next, the phase of the filtered LFP signal at each time step *θ*(*t*) is extracted using the Hilbert transform. Instantaneous frequency *f*(*t*) is then computed from the phase advance between each pair of adjacent samples and smoothed with a 50 ms box‐car filter. Finally, dynamic phase and frequency information are down‐sampled using linear interpolation to match the simulation time step of 5 ms (i.e., 200 Hz), and time windows that match the duration of tracking data for each run *T* are selected at random from randomly chosen electrode contacts. Importantly, there is no correspondence between these tracking data and the human LFP data used in each simulation, although both location (derived from the former) and LFP phase (derived from the latter) jointly determine the grid cell phase code according to Equations (3) and (4).

### 2.5 Grid cell analysis

We restrict all grid cell analyses to periods of movement, defined as time bins where running speed $\left\|\vec{v}\right\|$ ≥ 5 cm s^−1^. This is intended to match standard hippocampal electrophysiology analysis protocols, which typically exclude data from low running speeds because place and grid cells exhibit non‐local coding during periods of immobility (Olafsdottir, Bush, & Barry, 2018).

First, we examine the grid cell firing rate code for location. For both 1D and 2D environments, we compute the mean firing rate in 2‐cm sided bins and then smooth with a five bin boxcar kernel. Grid fields are subsequently defined as at least 5/10 contiguous bins in 1D/2D environments where firing rates are greater than 10% of the peak firing rate across the entire trial. For 2D environments, we quantify the heterogeneity of peak in‐field firing rates for each cell using the coefficient of variation, which is equal to the standard deviation of peak firing rates across fields divided by their mean (Ismakov et al., 2017). In addition, rate maps are used to generate spatial autocorrelations from which gridness scores and grid scale can be estimated, as described previously (Sargolini et al., 2006). To establish whether a rate map shows significant six‐fold symmetry, the true gridness is compared with the 99th percentile of a surrogate distribution generated by rotating the spike train relative to the tracking data by a time shift sampled from a random uniform distribution in the range *t*_shuf=_[1 : *T* − 1]s.

Second, we examine the temporal dynamics of grid cell firing. We estimate the phase modulation of each simulated spike train by the LFP using the resultant vector length of the circular distribution of firing phases. To establish whether simulated grid cells show significant phase locking, the true resultant vector length is compared with the 99th percentile of a surrogate distribution generated by rotating the spike train relative to the LFP signal by a time shift sampled from a random uniform distribution in the range *t*_shuf=_[1 : *T* − 1] s. Next, we generate spike time autocorrelations across a window of *t*_AC, win_=2 s with time bins of *t*_AC, bin_=10 ms and use these to estimate an oscillation index for each cell (following Eliav et al., 2018; Royer, Sirota, Patel, & Buzsáki, 2010). This is achieved by setting the value of the autocorrelation at zero lag to the maximum value across all time lags *t* and then fitting the corrected autocorrelation with a function *A*(*t*):

(7) $$A\left(t\right)=a\;\exp\left(-\frac{t}{\tau_{1}}\right)\left(\cos\left(2\mathrm{\pi Ft}\right)+1\right)+b\;\exp\left(-\frac{t}{\tau_{2}}\right)+c\;\exp\left(-\frac{t^{2}}{\tau_{3}^{2}}\right)+d.$$

Fitted parameter values are restricted to the following ranges: *a*, *b*, *d* = [0, *m*], *c* = [–*m*, *m*], *F* = [2, 20] Hz, *τ*_1_, *τ*_2_ = [0.1, 100] s, and *τ*_3_ = [0, 0.05]s; where *m* is the maximal value of the autocorrelation. Nonlinear least squares fitting is performed 500 times using the Matlab “fit” function with different initial values drawn randomly from a uniform distribution within the ranges specified above, and the estimated values of each parameter taken from the fit with the greatest *R* ^2^ value compared to the true autocorrelation. The oscillation index is then computed as *a*/max(*A*(*t*)).

To compute the instantaneous frequency difference between simulated grid cell spikes trains and the LFP, we estimate the oscillatory phase of the mean normalized grid cell phase code *r*_*ϕ*_(*ϕ*, *θ*) at each time step using the Hilbert transform, compute instantaneous frequency as the change in phase between time steps, and then compare this to the LFP frequency within the same time bin. Finally, phase precession is quantified as the circular‐linear correlation between firing phase and distance travelled through the grid field (Jeewajee, Barry, et al., 2008a; Kempter, Leibold, Buzsáki, Diba, & Schmidt, 2012). The significance of this correlation is established by randomly shuffling the firing phase values relative to the distance values 1,000 times, re‐computing the value of the circular‐linear correlation each time and then comparing the true correlation value to this surrogate distribution.

### 2.6 Decoding

Having simulated grid cell population activity, we then attempt to reconstruct the running speed, location and movement direction of our simulated agent, as well as an arbitrary fourth variable, from grid cell population firing patterns within each oscillatory cycle. To do so, we first compute the total number of spikes fired by each cell$\;\vec{k_{i}}$ in oscillatory cycle *i* (i.e., the *N* dimensional population vector); as well as the average location $\vec{x_{i}}$ and running speed $\left\|\vec{v_{i}}\right\|$ of the simulated agent in that cycle.

We then decode running speed as follows: we estimate the slope and intercept of the relationship between the total number of spikes fired (i.e., the sum of population vector $\vec{k_{i}}$ across all cells) and running speed in each cycle, using data from alternate cycles to avoid overfitting. We predict running speed in the remaining oscillatory cycles based on the total number of spikes fired by the grid cell population, and quantify decoding accuracy by comparing predicted and actual running speed values.

We then decode location, irrespective of firing phase, for all cycles with an average running speed ≥5 cm s^−1^ using maximum likelihood estimation (following Mathis et al., 2012). To do so, we compare the population vector $\vec{k_{i}}$ from cycle *i* with the expected firing rate in each location$\;\bar{r_{x}}\left(\vec{x}\right)$, independent of phase coding, produced by averaging the grid cell rate for location $r_{x}\left(\vec{x}\right)$ across 2 cm sided bins covering the entire environment, multiplied by the average cycle duration $\bar{T_{\mathrm{cyc}}}$. The maximum likelihood estimate of the agent's location given the population vector from that cycle ${\vec{x}}_{\mathrm{MLE}}\left(\vec{k_{i}}\right)$ is then given by:

(8) $${\vec{x}}_{\mathrm{MLE}}\left(\vec{k_{i}}\right)=\max_{\vec{x}}\left\{\prod_{N}\frac{{\left(\bar{r_{x}}\bar{T_{\mathrm{cyc}}}\right)}^{k_{i}}}{k_{i}!}\exp\left(-\bar{r_{x}}\bar{T_{\mathrm{cyc}}}\right)\right\}.$$

We decode location, incorporating phase information, as follows: we divide each oscillatory cycle *i* into *n*_*φ*_ = 5 phase bins with approximately equal spike counts (again, excluding cycles with an average running speed <5 cm s^−1^) and compute the *N* × *n*_*φ*_ dimensional population vector $\vec{k_{i,\varphi }}$ (i.e., the total number of spikes fired by each cell in each phase bin of each oscillatory cycle). We then compute the expected number of spikes in each phase bin of each cycle based on the combined rate and phase code for location$\;\bar{r_{g}}$ multiplied by the average phase bin duration$\;\bar{T_{\varphi }}$. As a control, to quantify the specific contribution of phase information, we also compute the expected number of spikes in each phase bin of each cycle based on the rate code for location alone$\;\bar{r_{x}}$ multiplied by the average phase bin duration$\;\bar{T_{\varphi }}$. The maximum likelihood estimate of the oscillatory cycle that produced the observed population vector $i_{g}\left(\vec{k_{i,\varphi }}\right)$ based on a combined rate and phase code is then given by:

(9) $$i_{g}\left(\vec{k_{i,\varphi }}\right)=\max_{i}\left\{\prod_{N,\varphi }\frac{{\left(\bar{r_{g}}\bar{T_{\varphi }}\right)}^{k_{i,\varphi }}}{k_{i,\varphi }!}\exp\left(-\bar{r_{g}}\bar{T_{\varphi }}\right)\right\}.$$

In addition, the maximum likelihood estimate of the oscillatory cycle that produced the observed population vector $i_{x}\left(\vec{k_{i,\varphi }}\right)$ based on a pure rate code is given by:

(10) $$i_{x}\left(\vec{k_{i,\varphi }}\right)=\max_{i}\left\{\prod_{N,\varphi }\frac{{\left(\bar{r_{x}}\bar{T_{\varphi }}\right)}^{k_{i,\varphi }}}{k_{i,\varphi }!}\exp\left(-\bar{r_{x}}\bar{T_{\varphi }}\right)\right\}.$$

In both cases, the decoded location is taken as the average location in the decoded oscillatory cycle. Importantly, in the presence of stable variation in peak in‐field firing rates, we can construct a “naïve” decoder by setting *r*_*c*_ = 1 when computing $\bar{r_{g}}$ and $\bar{r_{x}}$ above; and an “informed” decoder by setting *r*_*c*_ to the values used to generate grid firing patterns for each cell.

Finally, we decode movement direction as follows: we use maximum likelihood estimation to decode location independently within each phase bin of each oscillatory cycle as described above (i.e., using Equation (8), but replacing the *N* dimensional population vector $\vec{k_{i}}$ from cycle *i* with the *N* dimensional population vector $\vec{k_{i,p}}$ from phase bin *p* in cycle *i*). This generates a sequence of decoded locations ${\vec{x}}_{\mathrm{MLE},\varphi }=\left\{x_{\mathrm{MLE},\varphi },y_{\mathrm{MLE},\varphi }\right\}$ within each oscillatory cycle. We compute the slope of *x*_MLE, *φ*_ and *y*_MLE, *φ*_ across phase bins using linear regression, and estimate movement direction as the inverse tangent of those slopes. The decoded movement direction can be compared with the true movement direction computed from the actual location in each phase bin $\vec{\;x_{i,\varphi }}=\left\{x_{i,\varphi },y_{i,\varphi }\right\}$ using the same method.

### 2.7 Empirical tests of phase coding

We also use our simulated data to examine how phase coding might be identified in typical empirical data sets consisting of spike times and an aperiodic LFP signal. To do so, we examine spike triggered LFP averages at different locations within the firing field, consistent with previous studies (Eliav et al., 2018). Importantly, however, we wish to eliminate any differences in amplitude between cycles, which can skew spike triggered LFP averages and thus reduce the sensitivity of this approach. We therefore generate a synthetic LFP signal *s*(*t*) with constant amplitude from the phase of the filtered LFP signal at each time step *θ*(*t*):

(11) $$s\left(t\right)=\cos\theta \left(t\right).$$

We then compute the average of this synthetic LFP signal in three equally sized regions of each firing field for each cell, extract the circular mean phase at the time of firing across fields and cells for analysis purposes, and the average synthetic signal across fields and cells for illustration purposes.

### 2.8 Approximating the LFP signal

Finally, we examine whether population spiking activity could provide a good substitute for the LFP baseline signals used in these simulations. To do so, we sum the firing rates of all simulated grid cells in each temporal bin and then band‐pass filter that multi‐unit activity using the same 2–20 Hz second‐order Butterworth filter applied to the true LFP signal (see above). In this case, however, we make use of a causal filter to approximate a biologically realistic leaky integration process (i.e., which can only depend on past, and not future, inputs). We then correlate filtered multi‐unit activity with the synthetic LFP signal *s*(*t*), to avoid confounds arising from dynamic changes in amplitude.

### 2.9 Data availability

Data available on request from the authors. Code for all simulations, along with sample LFP data, is available at http://modeldb.yale.edu/261878.

## 3 RESULTS

### 3.1 Grid cell phase coding in the absence of rhythmicity

In contrast to the rodent, prominent and sustained oscillatory activity in LFP recordings from the human brain are rare. This has led to the suggestion that phase coding cannot play a role in human cognition, despite the numerous theoretical advantages afforded by such a scheme. Here, using intracranial LFP recordings from the human hippocampus, we aim to demonstrate that a robust phase code can be maintained in the absence of a prominent baseline oscillation with relatively fixed frequency; and to examine the functional advantages offered by phase coding over and above a pure rate code. As a model system, we make use of grid cells, which are characterized by a regular triangular array of spatial firing fields (Hafting, Fyhn, Molden, Moser, & Moser, 2005). In the rodent, grid cell firing is also modulated by the movement related 6–12 Hz LFP theta oscillation and a significant proportion of grid cells exhibit theta phase precession—firing at successively earlier phases of each theta cycle as their firing field is traversed (Climer et al., 2013; Hafting et al., 2008; Jeewajee et al., 2014). In humans and flying bats, however, grid cells appear to exist in the absence of a prominent LFP oscillation (Eliav et al., 2018; Jacobs et al., 2013; Yartsev et al., 2011).

**Figure 1:**
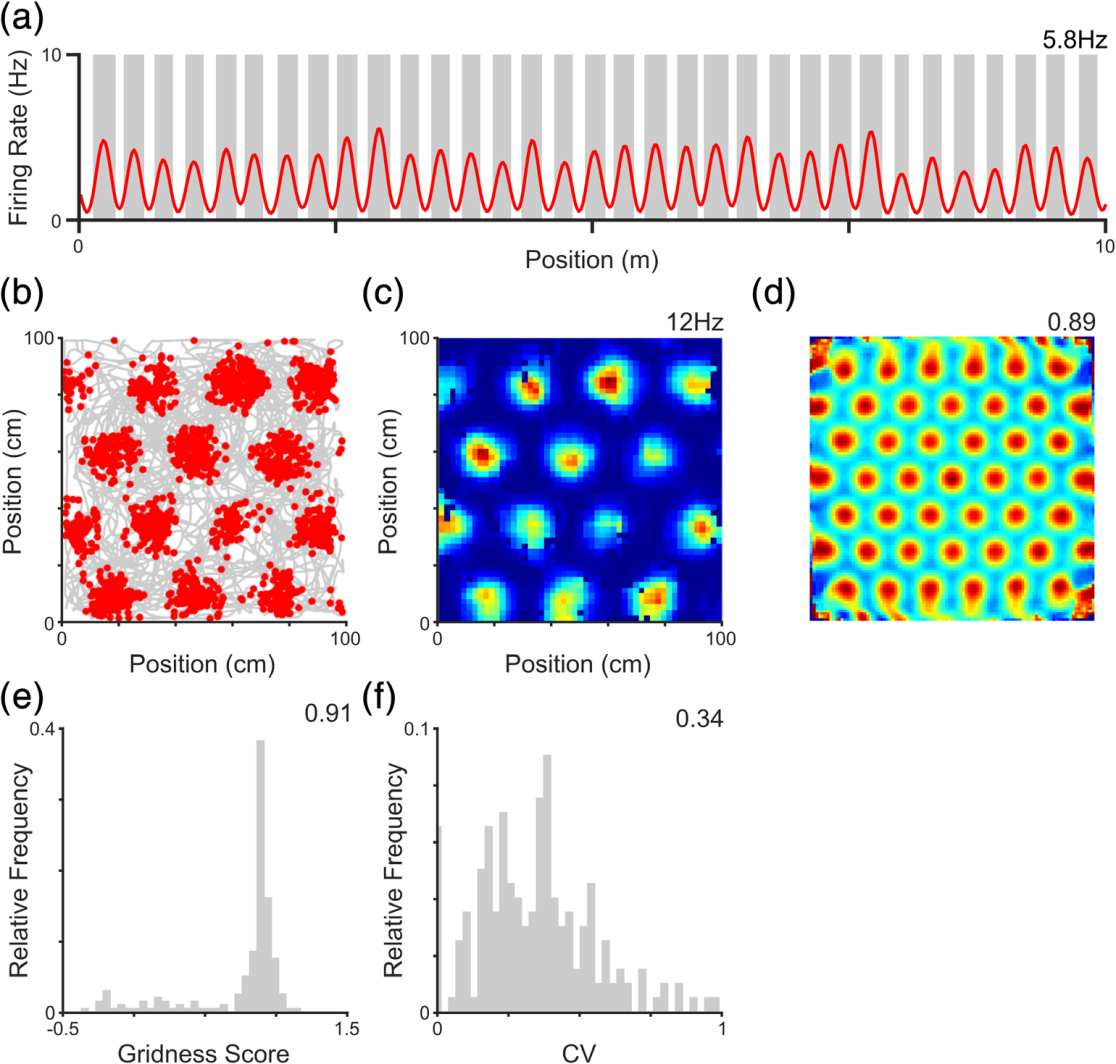
The grid cell rate code. As observed in vivo, simulated grid cells exhibit a firing rate code for location that corresponds to periodic spatial firing fields both (a) on a linear track; and (b) in the open field. This is confirmed by an inspection of the (c) firing rate map (peak rate inset); and (d) spatial autocorrelation (gridness score inset). (e) Distribution of gridness scores across the population, which are significant for all cells (median value inset). Lower gridness scores are produced by larger scale grids, which typically exhibit only two firing fields within the environment. (f) Finally, grid cells show substantial variation of peak in‐field firing rates, quantified using the coefficient of variation (CV) for each cell (median value inset). Panel a shows data from a single simulated grid cell, averaged over 20 independent simulations; panels b–d show data for a single simulated grid cell from a single simulation; panels e and f for the entire grid cell population from a single simulation [Color figure can be viewed at wileyonlinelibrary.com]

**Figure 2:**
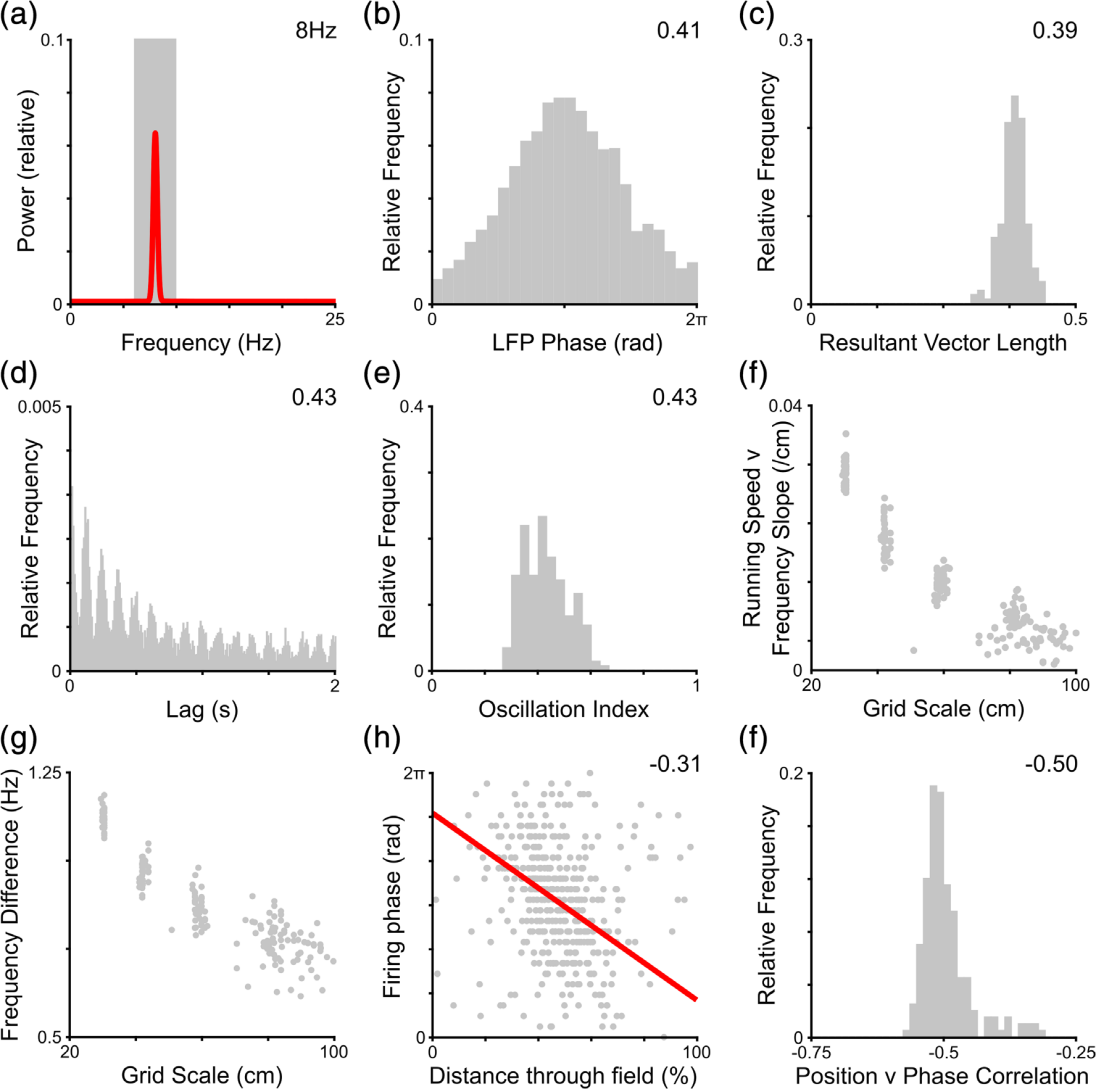
The grid cell temporal code (all data from simulations in the two‐dimensional [2D] environment). (a) Consistent with empirical data from rodent grid cells, the local field potential (LFP) signal in our initial simulations consists of a constant 8 Hz theta oscillation that is visible as a prominent peak in the power spectrum (6–10 Hz theta band marked in gray). (b) The firing rate of individual grid cells is modulated by LFP phase, firing preferentially at the trough of the theta oscillation (resultant vector length inset). (c) Distribution of firing phase resultant vector lengths across the population, which are significant for 99% of cells (median value inset). (d) Temporal autocorrelation of a typical grid cell, illustrating strong oscillatory activity in the theta band (oscillation index inset). (e) Distribution of oscillation indices across the population (median value inset). (f) The slope of the running speed versus grid cell burst firing frequency relationship is positive for all grid cells and inversely proportional to grid scale. (g) As a result, the intrinsic firing frequency of individual grid cells exceeds the LFP frequency by an amount that is inversely proportional to their grid scale. (h) This is consistent with phase precession at the single cell level, visible as a negative correlation between distance through the firing field and firing phase (circular‐linear correlation coefficient inset, and line of best fit plotted in red). (i) Distribution of circular‐linear correlation coefficients across the grid cell population, which are significant for all cells (median value inset). Panel a shows data from a single simulation; panels b, d, and h for a single simulated grid cell; panels c, e–g, and i for the entire grid cell population [Color figure can be viewed at wileyonlinelibrary.com]

We begin by simulating the firing patterns of rodent grid cells using a phenomenological model in which the influence of location on both firing rate and preferred firing phase can easily be modulated (Chadwick et al., 2015; see Section 2). An examination of firing rates confirms that simulated grid cells show periodic spatial firing patterns in both 1D (Figure 1a) and 2D (Figure 1b–d) environments. This periodicity can be quantified using the gridness metric, which is significant for all cells in the 2D environment, but lower for larger scale grids that exhibit fewer fields within the arena (Figure 1e). Interestingly, simulated grid cells also exhibit substantial variation of peak in‐field firing rates, despite these simulations being intended to produce a uniform grid firing pattern (Figure 1f). This is likely to result from Poisson firing statistics and non‐uniform coverage of the environment, consistent with previous theoretical studies (Ismakov et al., 2017).

As in the rodent entorhinal cortex, grid cell firing in these initial simulations is accompanied by a prominent ~8 Hz theta oscillation in the LFP (Figure 2a). The firing rate of individual cells is strongly modulated by the ongoing theta rhythm, both at the single cell (Figure 2b) and population level (Figure 2c), with 99% of cells showing significant phase modulation. Indeed, oscillatory activity in the theta frequency band is visible in the temporal autocorrelation of single unit firing (Figure 2d) and demonstrated by high oscillation indices across the population (Figure 2e), consistent with rodent experimental data (Eliav et al., 2018). Importantly, however, the burst firing frequency of grid cells is slightly higher than LFP theta, and this difference increases with running speed at a rate that is inversely proportional to the measured grid scale (Geisler et al., 2007, 2010; Figure 2f). As a result, the average frequency difference between the burst firing of grid cells and the LFP across the entire simulation is inversely proportional to grid scale (Figure 2g; Jeewajee, Barry, et al., 2008a). Hence, grid cells across the population exhibit theta phase precession—firing progressively earlier in each theta cycle as their firing fields are traversed (Figure 2h,i).

**Figure 3:**
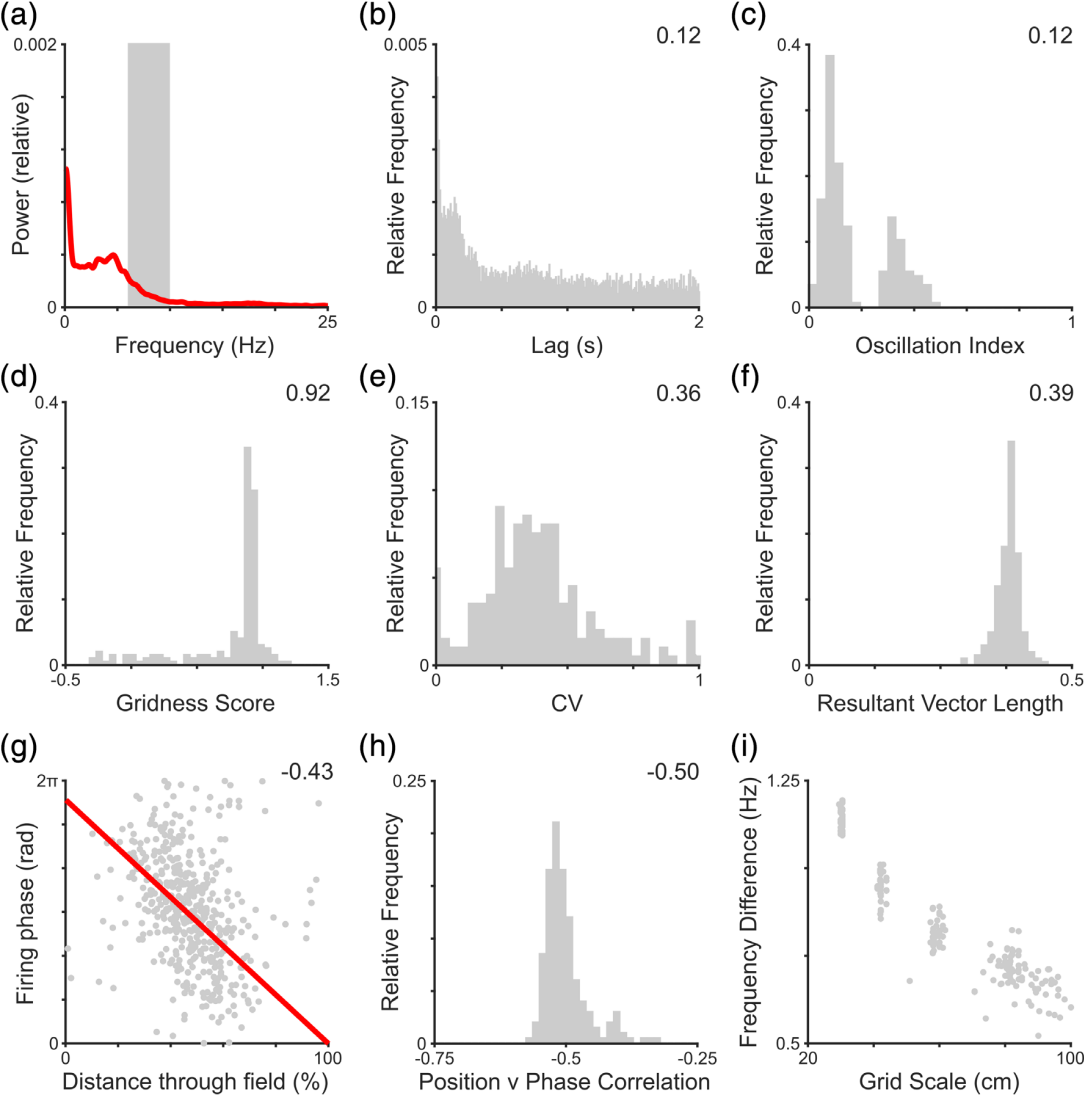
The grid cell temporal code in the absence of rhythmicity. In these simulations, we make use of a local field potential (LFP) signal recorded from the human hippocampal formation with a frequency that varies dynamically over a broad range, such that no clear peak is observed in either the: (a) LFP power spectrum (6–10 Hz theta band marked in gray); or (b) spike train temporal autocorrelation (oscillation index inset). (c) Distribution of oscillation indices, which are significantly lower than simulations with constant theta rhythmicity (median value inset). (d) Distribution of gridness scores across the population, which are significant for 99.5% of cells and do not differ from simulations with constant theta rhythmicity (median value inset). (e) Distribution of peak in‐field firing rate coefficient of variation (CV) values, which do not differ from simulations with constant theta rhythmicity (median value inset). (f) Distribution of firing phase resultant vector lengths across the population, with 99% of cells showing significant phase modulation (median value inset). (g) Correlation between distance travelled through the grid field and firing phase for a typical grid cell, illustrating phase precession (circular‐linear correlation coefficient inset, and line of best fit plotted in red). (h) Distribution of circular‐linear correlation coefficients across the population, which are significant for all cells and do not differ significantly from those with constant theta rhythmicity (median value inset). (i) The intrinsic firing frequency of individual grid cells continues to exceed the LFP frequency by an amount that is inversely proportional to their grid scale, despite that frequency varying dynamically over a wide range. Panel a shows data from a single simulation; panels b and g for a single simulated grid cell; panels c–f, h, and i for the entire grid cell population [Color figure can be viewed at wileyonlinelibrary.com]

Next, we simulate the firing pattern of grid cells recorded in the human or flying bat brain where prominent, sustained oscillations are rare (Eliav et al., 2018; Jacobs et al., 2013; Yartsev et al., 2011). To do so, we make use of LFP recordings extracted from depth electrodes in the hippocampal formation of pre‐surgical epilepsy patients performing a spatial memory task in place of a constant 8 Hz theta oscillation (Bush et al., 2017). In this case, LFP frequency varies dynamically over a broad range, such that no clear peak is observed in either the power spectrum (Figure 3a) or temporal auto‐correlogram of simulated spike trains (Figure 3b). At the population level, this is confirmed by a significant reduction in grid cell oscillation indices (*t*[398] = −23.6, *p* < 0.001, Cohen's *d* = 2.36; Figure 3c, compare with Figure 2e), consistent with data from flying bats (Eliav et al., 2018). Nonetheless, the firing rate code for location provided by grid cells is preserved, as demonstrated by the distribution of gridness scores from the 2D environment (Figure 3d), which remain significant for 99.5% of cells and do not differ significantly from simulations with constant theta rhythmicity (*t*[398] = 0.48, *p* = 0.63; compare with Figure 1e). Similarly, the coefficient of variation in peak in‐field firing rates does not differ from simulations with constant theta rhythmicity (*t*[398] = 1.56, *p* = 0.12; Figure 3e, compare with Figure 1f).

Crucially, the firing of individual cells in these simulations remains modulated by the phase of low frequency fluctuations in the LFP (Figure 3f), with no significant change from simulations with constant theta rhythmicity (*t*[398] = −1.52, *p* = 0.13; compare with Figure 2c). Moreover, simulated cells exhibit phase precession in the absence of spike train rhythmicity, showing a negative correlation between the phases of low frequency LFP oscillations at which firing occurs and distance travelled through the firing field at the single cell level (Figure 3g). Indeed, there is no significant difference in location versus firing phase correlation coefficients across the population (Figure 3h) compared to simulations with constant theta rhythmicity (*t*[398] = −1.14, *p* = 0.26; compare with Figure 2i). Finally, the average difference between the intrinsic firing frequency of grid cells and instantaneous LFP frequency also remains inversely proportional to grid scale, despite the LFP frequency varying dynamically over a wide range throughout navigation (Figure 3i; compare with Figure 2g).

**Figure 4:**
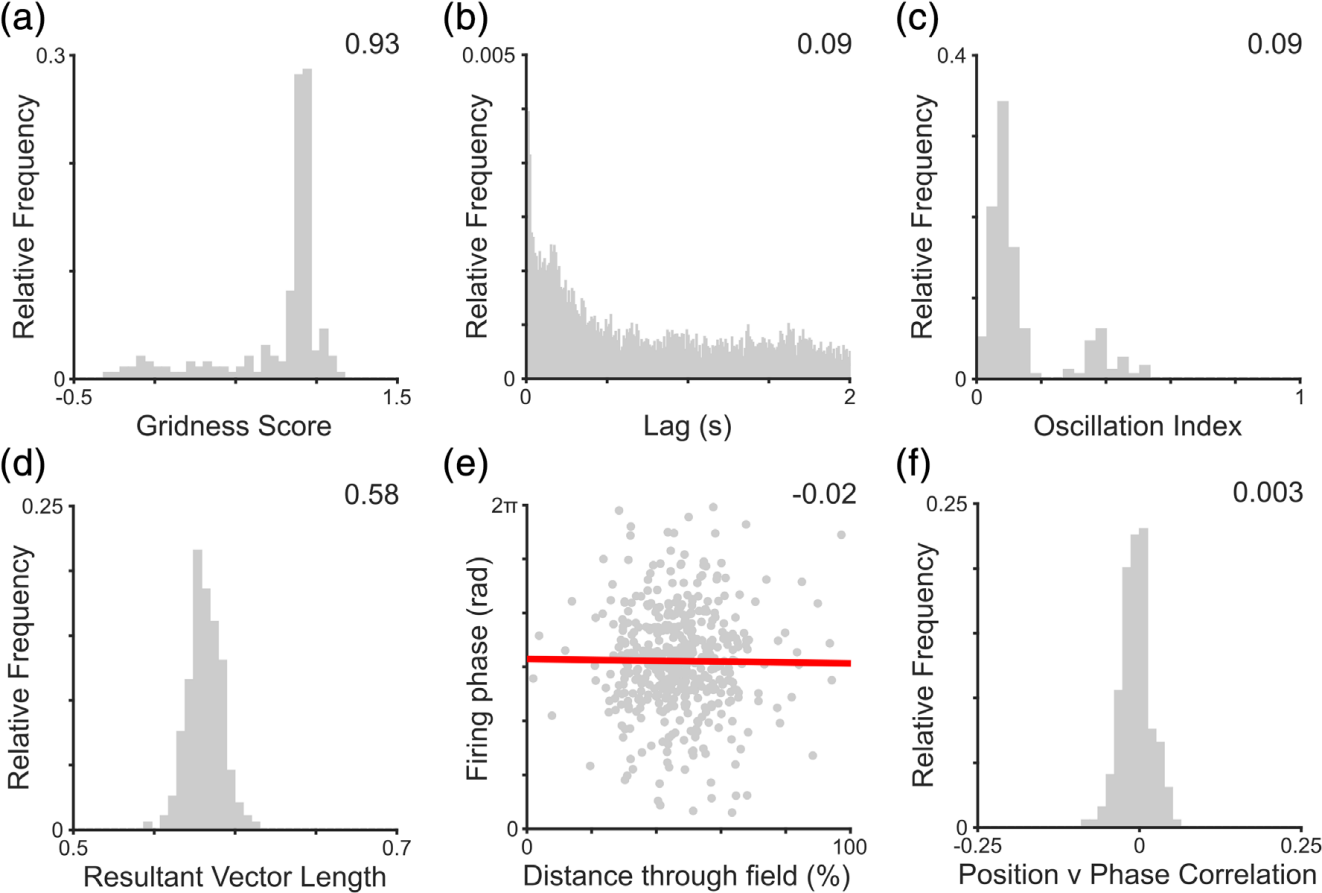
Phase locked grid firing in the absence of rhythmicity. (a) Distribution of gridness scores across the population, which are significant for all cells and do not differ from simulations with phase precession (median value inset). (b) Sample spike train temporal autocorrelation, indicating the absence of rhythmicity in simulated grid cell firing (oscillation index inset). (c) Distribution of oscillation indices across the population, indicating the absence of prominent theta rhythmicity in grid cell spike trains (median value inset). (d) Distribution of firing phase resultant vector lengths, which are significant for all cells and significantly higher than simulations with phase precession (median value inset). (e) Correlation between distance travelled through the grid field and firing phase for a typical grid cell, illustrating the absence of phase precession (circular‐linear correlation coefficient inset, and line of best fit plotted in red). (f) Distribution of location versus phase correlation coefficients across the population, which are significant for only 5% of cells and significantly higher than simulations with phase precession (median value inset). Panels b and e show data for a single simulated grid cell; panels a, c, d, and f for the entire grid cell population [Color figure can be viewed at wileyonlinelibrary.com]

In each of the simulations described above, all grid cells exhibit phase precession—that is, their preferred firing phase is dictated by progress through the firing field (see Section 2). However, in empirical data from both rodents (Climer et al., 2013; Hafting et al., 2008; Jeewajee et al., 2014; Sargolini et al., 2006) and flying bats (Eliav et al., 2018), a significant proportion of grid cells also exhibit phase locking—consistently firing at the same phase of low frequency fluctuations in the LFP. Hence, we next simulate grid cell firing that is phase locked to the highly variable human intracranial LFP signal, as these data will later serve as a useful control to establish the specific functional contribution offered by phase coding. In these simulations, gridness scores remain significant for all cells (Figure 4a) and do not differ significantly from previous simulations using human LFP data (*t*[398] = 0.02, *p* = 0.98; compare with Figure 3d). In the presence of a variable frequency baseline oscillation, there is no clear peak in the spike train auto‐correlogram (Figure 4b) and oscillation indices across the population remain low (Figure 4c). Nonetheless, all cells exhibit significant phase locking values (Figure 4d) that exceed those in previous simulations using human LFP data, which exhibited phase precession (*t*[398] = 115.0, *p* < 0.001, *d* = 11.5; compare with Figure 3f). Conversely, no phase precession is observed in these simulations, either at the single cell level (Figure 4e) or across the population (Figure 4f), with location versus phase correlation coefficients being significantly higher than those in previous simulations using human LFP data (*t*[398] = 152.7, *p* < 0.001, *d* = 15.3; compare with Figure 3h, noting the change in *x*‐axis) and not different from zero (*t*[199] = 0.80, *p* = 0.43).

In sum, these results demonstrate that both the phase precession and phase locking of grid cell firing could occur in the absence of any apparent rhythmicity in either the LFP or spike train, given that phase is independent of frequency (Blair et al., 2013; Orchard, 2015). This is consistent with recent empirical data from bats, where place and grid cells show either phase locking or phase precession relative to an LFP signal with a frequency that varies dynamically over a wide range (Eliav et al., 2018). Importantly, this also demonstrates that LFP dynamics in the human hippocampus do not preclude the existence of robust phase coding (see Section 4 for potential mechanisms). Nonetheless, it should be emphasized that none of the results presented here are contingent on specific features of those LFP data. Any baseline signal with broadband low frequency power from which phase information can be extracted after filtering (here, in the 2–20 Hz range) would produce the same qualitative output, regardless of whether that signal exhibited rhythmicity within a specific, narrow frequency range. Indeed, multi‐unit activity in these simulations provides a good substitute for the LFP signal (see Section 2 and Figure 7d,e). It is also important to note that there is no correspondence between the rodent behavioral trajectories and human LFP data used in these simulations, although both location (derived from the former) and LFP phase (derived from the latter) jointly determine the grid cell phase code for location. Next, we examine the potential functional contribution made by that phase code in grid cells; and how this might be affected by the absence of a prominent baseline oscillation with approximately constant frequency.

### 3.2 Grid cells multiplex spatial information in firing rate and phase

**Figure 5:**
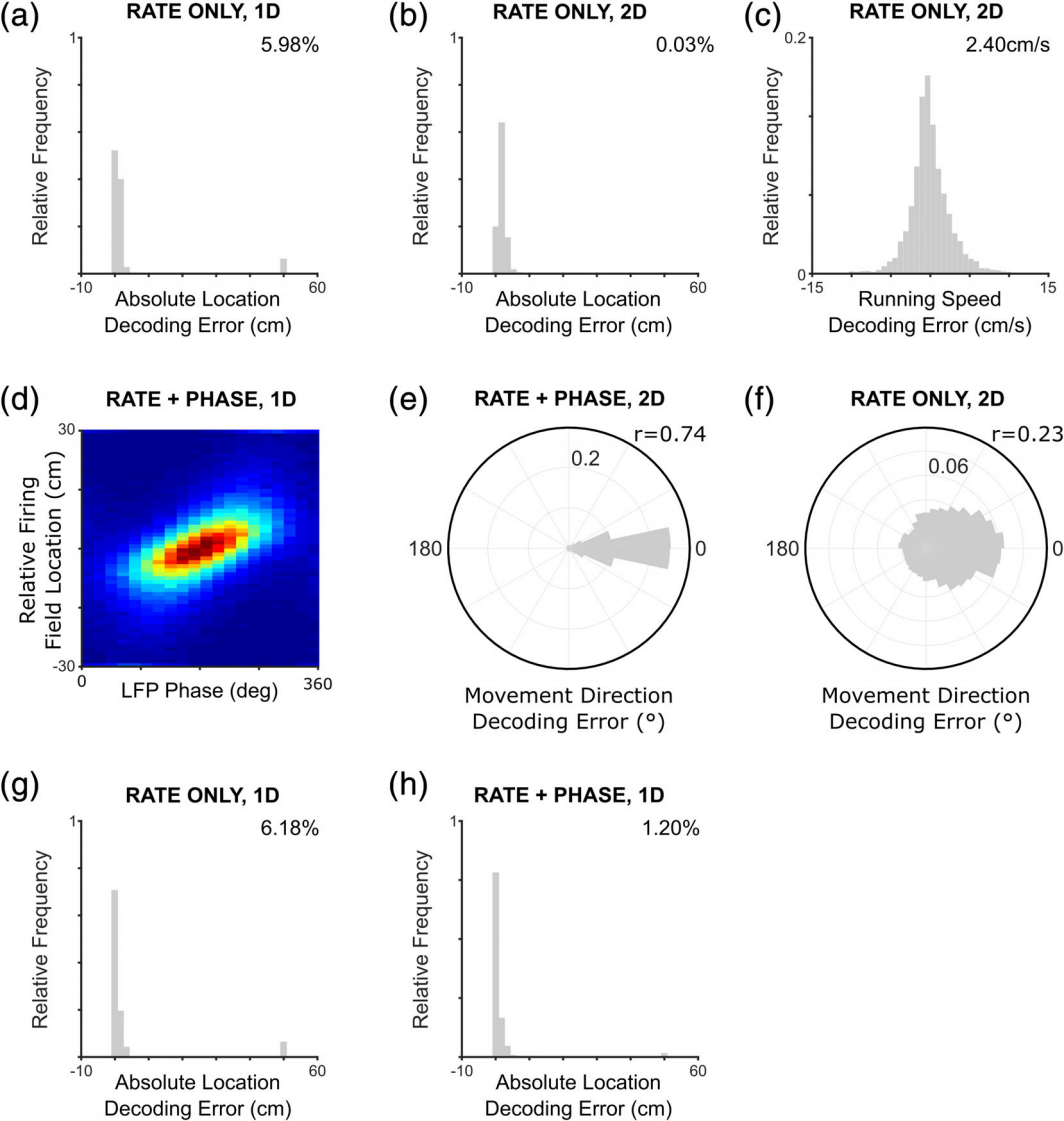
Decoding movement trajectories from grid cell population activity in each oscillatory cycle. Location in both (a) one‐dimensional (1D) and (b) two‐dimensional (2D) environments can be decoded from population firing rates, using the grid cell rate function (frequency of catastrophic errors inset). (c) In addition, running speed can be decoded from the total number of spikes fired by the grid cell population (standard deviation inset). (d) Phase precession across the grid cell population generates “theta sequences” of activity within each oscillatory cycle that correspond to the current movement trajectory (color axis indicates spike density). (e) This allows movement direction to be estimated from the sequence of locations decoded across phase bins (resultant vector length inset). (f) Conversely, in the absence of a phase code for location, movement direction decoding is much less accurate (resultant vector length inset). (g) Location can also be decoded from population firing rates, using the history of firing rates across oscillatory cycles in that simulation (frequency of catastrophic errors inset). (h) However, decoding location using both population firing rate and phase information is more accurate, as illustrated by a significant reduction in the rate of catastrophic errors (frequency of catastrophic errors inset). Panels a, b, g, and h are averaged over data from 20 independent simulations [Color figure can be viewed at wileyonlinelibrary.com]

To address the potential contribution made by phase coding to cognition, we next examine what information can be decoded from grid cell population activity within each oscillatory cycle and how this is affected by the presence or absence of a phase code for location within the firing field. Several previous theoretical studies have established that, while the firing pattern of a single grid cell or module of grids cells is inherently ambiguous about an animal's location, grid cell population activity across modules with different scales can offer a robust and accurate code for location and navigation over a large range (Bush, Barry, Manson, & Burgess, 2015; Fiete et al., 2008; Mathis et al., 2012; Stemmler, Mathis, & Herz, 2015). Consistent with those studies, we find that even the relatively small population examined here (five modules, containing a total of 200 grid cells) can encode location with a median error of <2 cm in both 1D (Figure 5a) and 2D (Figure 5b) environments. In the larger (~50 m) 1D environment, however, the grid cell rate code is prone to occasional catastrophic errors (defined here as ≥50 cm), consistent with previous studies (Fiete et al., 2008; Mathis et al., 2012; Towse, Barry, Bush, & Burgess, 2014). These large errors reflect the upper capacity limit of the grid cell network, which is generally dictated by the lowest common multiple of grid scales (Bush et al., 2015; Fiete et al., 2008; but see Mathis et al., 2012; Stemmler et al., 2015).

In addition to encoding location, empirical data indicate that the mean firing rate of grid cells—like those of other principal cells in the hippocampal formation—varies with running speed (Sargolini et al., 2006). The cells simulated here also exhibit this property (see Section 2), such that we can estimate the agent's running speed from the total number of spikes fired by the grid cell population within each oscillatory cycle (as demonstrated previously for speed cells; Gois & Tort, 2018; Kropff, Carmichael, Moser, & Moser, 2015). This allows us to decode running speed with an accuracy of ≤5 cm s^−1^ in 95% of cycles (Figure 5c). Hence, these results demonstrate that both location and movement speed can be accurately decoded from the firing rate of a small number of grid cells in each oscillatory cycle and, importantly, that the modulation of firing rates according to running speed does not compromise location decoding.

Next, we turn to the nature of information encoded by the grid cell phase code, which has previously received relatively little attention. In place cells, it has been demonstrated that theta phase precession causes sweeps of activity corresponding to the animal's current trajectory to occur within each oscillatory cycle (Burgess et al., 1994; Johnson & Redish, 2007; Skaggs et al., 1996; Figure 5d). It has been suggested that movement direction might therefore be encoded by the relative phase of place or grid cell firing across the population (Zutshi et al., 2017), although this has yet to be demonstrated. Consistent with this hypothesis, we demonstrate that—by fitting a linear trajectory to the sequence of locations decoded independently from five phase bins within each oscillatory cycle—we can estimate movement direction with an accuracy of ≤30° in 76% of cycles (Figure 5e). In contrast, the decoding of movement direction is significantly less accurate in simulations without phase coding, where an accuracy of ≤30° is achieved in only 30% of cycles (Figure 5f), although still possible in some cases due to the small but significant movement made within each oscillatory period. Specifically, the distribution of movement direction decoding errors is non‐uniform with a mean of zero both with (*V* = 4,745, *p* < 0.001) and without (*V* = 1,742, *p* < 0.001) phase coding, although the variance is significantly greater in the latter case (*k* = 18.8 × 10^6^, *p* < 0.001). This demonstrates that the grid cell phase code for location contributes to the accurate encoding of movement direction in population activity within each oscillatory cycle.

Importantly, the decoding of movement direction from grid cell population activity does not require an explicit measure of grid cell firing phase—downstream neurons could simply read out a location estimate from simultaneously active grid cells in different phase bins by coincidence detection. Movement direction is subsequently estimated from the sequence of decoded locations across the oscillatory cycle, and each sequence of decoded locations is punctuated by a period of relatively low population firing rates between oscillatory cycles. Similarly, accurate decoding of movement direction does not require the phase of firing in individual grid cells to be particularly precise—only that firing phase can be measured. In these simulations, the combination of Poisson firing statistics and a relatively broad preferred firing phase distribution (e.g., with a circular standard deviation of ~0.9 rad, see Equation (3)) produce a significant level of phase noise in simulated grid cells. Moreover, the results described above are generated by independently decoding location from grid cell population activity in five phase bins, corresponding to a phase resolution of ~72° or 2π/5.

Elsewhere, previous studies have demonstrated that incorporating phase information can improve the accuracy with which location is decoded from place cell firing rates (Jensen & Lisman, 2000). To establish if this is also true for grid cell firing patterns, we compare the accuracy with which location can be decoded in the larger (~50 m) 1D environment. We find that incorporating the phase of firing significantly improves location decoding accuracy, compared to decoding using firing rates alone (Figure 5g,h). Specifically, incorporating phase information significantly reduces the incidence of catastrophic location decoding errors (*t*[38] = −12.0, *p* < 0.001, *d* = 3.79). Finally, we note that the frequency of the baseline oscillation in these simulations varies independently of grid cell firing rate and phase, and could therefore be used to encode an additional variable. In the rodent, for example, it has been demonstrated that LFP theta frequency encodes running speed information, like neural firing rates, and possibly driven by the same medial septal glutamatergic inputs (Fuhrmann et al., 2015; Hinman, Brandon, Climer, Chapman, & Hasselmo, 2016; Wells et al., 2013). Similarly, in the flying bat, it has been demonstrated that LFP frequency is higher during faster movements (Eliav et al., 2018). Although baseline frequency is not modulated by running speed in these simulations, we find that it is possible to accurately decode an arbitrary fourth variable from the duration of each oscillatory cycle by linear regression, with an error of ≤5% in 91% of cycles (data not shown).

In sum, these results demonstrate that the rate and phase code for location provided by grid cells can efficiently and accurately multiplex information about the complete movement trajectory of an agent, including location, running speed, and movement direction, in addition to a fourth variable provided by the oscillatory period. In the absence of phase coding, movement direction can no longer be accurately decoded from grid cell activity, and the accuracy of location decoding is reduced. Hence, phase coding in grid cells makes a specific contribution to the encoding of movement information above and beyond that provided by an examination of firing rates alone. Crucially, each of these results could equally arise from decoding place cell firing patterns, which exhibit an equivalent rate and phase code for location. Our decision to simulate grid cell firing patterns was driven solely by computational efficiency, as fewer grid cells are required to accurately encode location (Fiete et al., 2008) and inherently provide a greater number of runs through the firing field in each simulation.

### 3.3 Phase coding with variable in‐field firing rates

**Figure 6:**
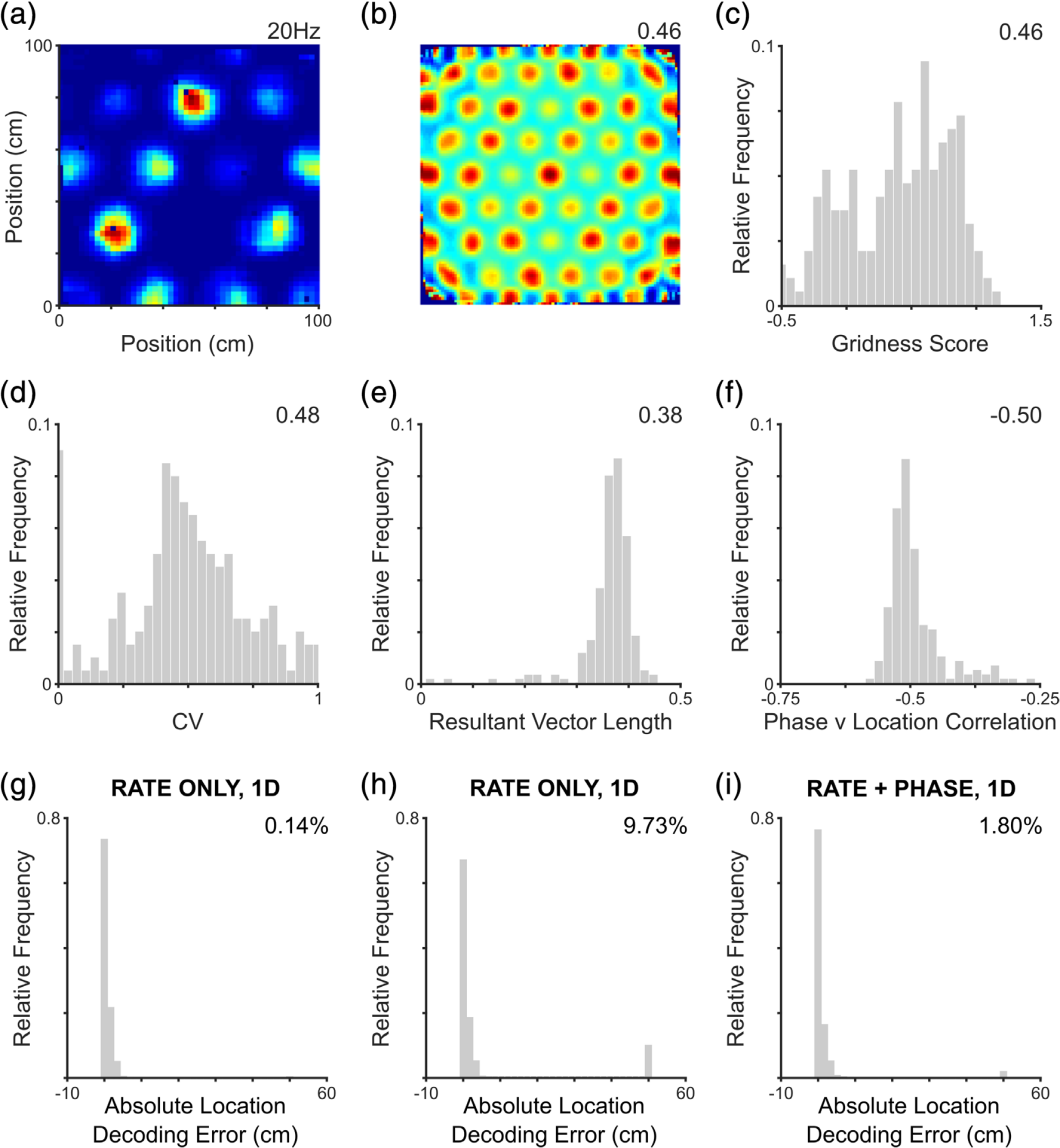
Phase contributes to accurate location decoding with variable in‐field firing rates. (a) Sample rate map from a simulated cell with stable differences in peak in‐field firing rate (overall peak firing rate inset). (b) Corresponding spatial autocorrelation (gridness score inset). (c) Distribution of gridness scores across the population, which are significant for 94% of cells but significantly lower than simulations with a uniform grid firing pattern (median gridness score inset); (d) Distribution of peak in‐field firing rate coefficient of variation (CV) values, which are significantly higher than simulations with a uniform grid firing pattern (median value inset); (e) Distribution of firing phase resultant vector lengths, which are significant for all cells (median value inset); and (f) Distribution of location versus phase correlation coefficients across the population, which are significant for all cells (median value inset). (g) Location decoding with an “informed” decoder. Accuracy is significantly improved when in‐field firing rates are variable and the decoder is informed about the expected firing rate in each field (compare with Figure 5g, frequency of catastrophic errors inset). (h) Location decoding with a “naïve” decoder. Accuracy is significantly worse when the decoder is naïve to the expected firing rate in each field (compare with Figure 5g, frequency of catastrophic errors inset). (i) Location decoding with a “naïve” decoder and phase information. Providing the naïve decoder with information about firing phase reduces ambiguity without the need for environment specific learning (frequency of catastrophic errors inset). Panels a and b show data for a single simulated grid cell; panels c–f for the entire grid cell population; and panels g, h, and i are averaged over data from 20 independent simulations [Color figure can be viewed at wileyonlinelibrary.com]

Interestingly, it has been demonstrated that grid cells exhibit stable differences in peak in‐field firing rates across familiar environments (Ismakov et al., 2017), with grid fields that are closer to persistently rewarded locations generally being more active (Boccara et al., 2019; Butler et al., 2019). It has been suggested that this allows salient locations to be encoded more accurately by the grid cell population, although empirical proof is lacking. We therefore chose to examine the impact of firing rate variability on the accuracy with which location can be decoded from the grid cell population by simulating grid cells with stable differences in peak in‐field firing rate that approximate the variability observed in vivo (Ismakov et al., 2017). As expected, grid firing patterns in these simulations are less uniform (Figure 6a,b) and population gridness scores significantly lower than previous simulations using human LFP data (*t*[398] = −9.59, *p* < 0.001, *d* = 0.96; Figure 6c, compare with Figure 3d), although the vast majority of cells continue to exhibit significant six‐fold symmetry. The coefficient of variation in peak in‐field firing rates across the population is significantly greater than that produced by uneven sampling of a uniform grid firing pattern (*t*[398] = 5.76, *p* < 0.001, *d* = 0.58; Figure 6d, compare with Figure 3e). Importantly, however, the firing rates of all simulated grid cells continue to be significantly modulated by low‐frequency fluctuations in the LFP signal (Figure 6e) and exhibit phase precession (Figure 6f).

Next, we examine location decoding accuracy in simulations on the larger (~50 m) linear track. First, we find that decoding accuracy is indeed improved by increased variability in in‐field firing rates when the decoder is informed about this variability—that is, when the decoder makes use of the expected peak firing rate in each grid field (Figure 6g). Specifically, we find that the relative frequency of catastrophic location decoding errors is significantly reduced when the variability of in‐field firing rates is increased (*t*[38] = −14.8, *p* < 0.001, *d* = 4.68; compare with Figure 5g). This may be computationally expensive, however, as such a decoder would have to learn the distribution of peak firing rates across grid fields for each new environment. Conversely, a naïve decoder—which expects peak firing rates to be equal across all grid fields—does not require any learning in each new environment, but produces significantly more catastrophic location decoding errors when faced with variable in‐field firing rates (*t*[38] = 6.39, *p* < 0.001, *d* = 2.02; Figure 6h, compare with Figure 5g). In this case, firing rate variability introduces ambiguity into the grid cell rate code for space, as low firing rates may indicate either the periphery of a field with high peak firing rate or the center of a field with low peak firing rate.

Intuitively, this ambiguity might be resolved by incorporating phase information, which specifically indicates the location of an agent within the firing field. Indeed, we find that decoding location using a uniform firing rate function alongside information about the phase of firing produces fewer catastrophic errors than the naïve rate decoder (*t*[18] = −19.7, *p* < 0.001, *d* = 6.22; Figure 6i, compare with Figure 6h), consistent with the results described above (Figure 5g,h). Although the incidence of catastrophic errors remains slightly but significantly greater than that produced by an informed decoder (*t*[38] = 11.7, *p* < 0.001, *d* = 3.69; compare with Figure 6g), the agent does not need to learn the configuration of peak firing rates in each novel environment. This is consistent with the notion that grid cells support the generalization of structural regularities between contexts (Behrens et al., 2018). In sum, these results indicate that variable in‐field firing rates only contribute to more accurate encoding of location by grid cells if the decoding apparatus is informed of the expected variability; and that providing the decoding apparatus with access to phase information can ameliorate this issue by resolving location within the firing field. This highlights another potential contribution of phase coding to cognition: disambiguating stimuli (i.e., locations) that produce similar firing rates.

### 3.4 Empirical tests for phase coding in the absence of rhythmicity

**Figure 7:**
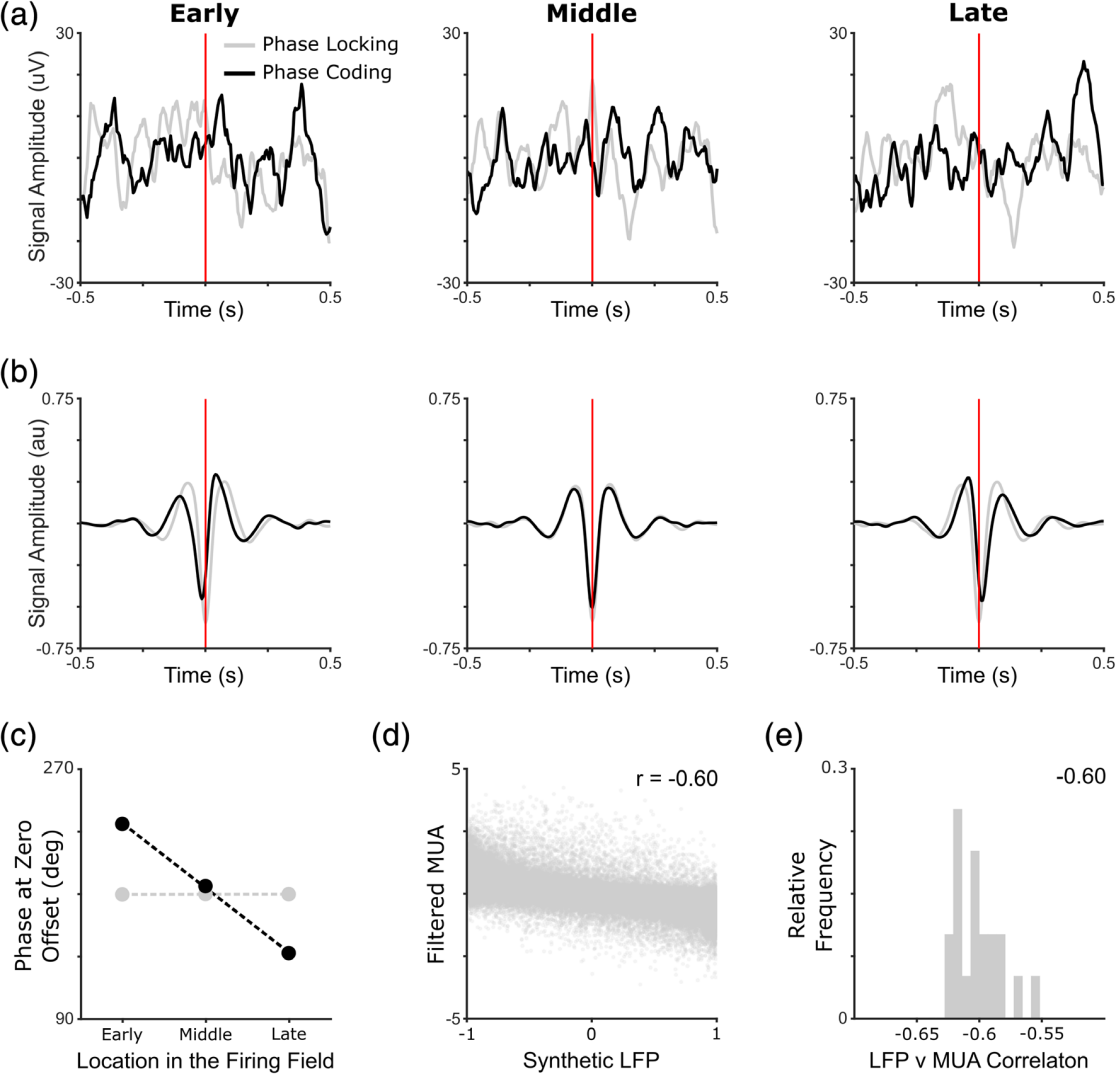
Empirical tests for phase coding in the absence of rhythmicity. (a) Spike triggered average of the local field potential (LFP) signal in early, middle and late parts of the firing field (one‐dimensional [1D] environment). (b) Spike triggered average of the same LFP signal, reconstructed from phase information after a broadband filter is applied, in early, middle and late parts of the firing field (1D environment). Peak deflection of the LFP signal progresses from a negative to positive time lag with progress through the receptive field, consistent with the presence of a phase code for location within the field. (c) Circular mean phase at the time of firing in early, middle and late parts of the firing field, averaged across all cells. Note that this is equivalent to the phase precession plots shown in Figures 2h and 3g, and can be statistically assessed by circular‐linear correlation analyses. (d) Correlation between synthetic LFP signal and filtered multi‐unit activity (MUA) in a representative 1D simulation (correlation coefficient inset). (e) Distribution of synthetic LFP versus filtered MUA correlation coefficients across 20 independent 1D simulations (median value inset, see Section 2 for details) [Color figure can be viewed at wileyonlinelibrary.com]

Finally, we consider the problem of how phase coding can be identified in empirical data when sustained rhythmicity is absent. One method is to examine spike‐triggered LFP averages. If LFP firing phase is modulated by some variable, such as distance through the receptive field or position within the spike train, then plotting the spike‐triggered LFP average for different values of that variable should reveal systematic variations. This approach has been successfully employed to reveal phase coding against an arrhythmic baseline signal in grid and place cells of the flying bat (Eliav et al., 2018). Complications arise, however, from the fact that LFP amplitude may vary independently of frequency, such that the LFP signal associated with spikes fired during periods of relatively high LFP amplitude can dominate spike‐triggered averages (Figure 7a). To avoid this potential confound, the baseline signal can be reconstructed from the cosine of its phase, which is extracted from the analytic signal after a broadband filter has been applied—effectively orthogonalizing phase information from variations in amplitude.

By using this approach on our simulated data, we find that spike‐triggered LFP averages clearly differ between early, middle and late parts of the grid firing field when simulated cells exhibit phase precession, but not when they exhibit phase locking (Figure 7b). Indeed, the circular mean phase at the time of spiking changes significantly across early, middle and late parts of the receptive field (Figure 7c), consistent with the phase precession analyses described above (e.g., Figures 2h and 3g). We emphasize that this approach is not specific to spatially modulated firing patterns—similar plots could be generated for different stimulus intensities, or positions within a spike train, to assay the presence of phase coding in a diverse range of cortical regions (e.g., Aghajan et al., 2015).

## 4 DISCUSSION

The simulations presented here support two important conclusions regarding the potential role of phase coding in human cognition. First, that phase coding does not necessarily rely on a prominent baseline oscillation with relatively invariant frequency, and thus that the absence of such a signal in typical LFP recordings from the human brain does not preclude a role for phase coding. This is consistent with recent empirical data from the flying bat, where a phase code for location is observed in place and grid cells of the hippocampal formation, despite the absence of any sustained oscillatory activity in a fixed frequency band (Eliav et al., 2018). Second, that phase coding allows for both the multiplexing of additional information in spike trains and the disambiguation of stimuli that generate similar firing rates, and thus offers computational advantages above and beyond those provided by firing rates alone. This is consistent with previous theoretical studies (Fries et al., 2007; Jensen et al., 2014; Panzeri et al., 2010; Thorpe et al., 2001) and empirical data (Kayser et al., 2009; Montemurro et al., 2008; O'Keefe & Burgess, 2005; Siegel et al., 2009; Turesson et al., 2012; Zuo et al., 2015), which demonstrate that phase modulation permits greater information content.

In the context of spatial cognition, we have demonstrated that phase coding within each oscillatory cycle encodes movement direction, consistent with previous suggestions (Zutshi et al., 2017). Movement direction cannot be reliably recovered from firing rates alone, although these can robustly encode both location and movement speed (Fiete et al., 2008; Mathis et al., 2012). Similarly, movement direction cannot be reliably extracted from the activity of head direction cells. This is demonstrated by experiments in which movement and head direction are set in opposition, showing that theta sequences continue to indicate the direction in which the animal is moving despite the head direction system encoding the exact opposite (Cei, Girardeau, Drieu, Kanbi, & Zugaro, 2014; Maurer, Lester, Burke, Ferng, & Barnes, 2014). Moreover, it has been demonstrated that very few—if any—single units in medial entorhinal cortex encode movement direction, despite such an input being essential to establish and maintain grid firing patterns and support path integration (Raudies, Brandon, Chapman, & Hasselmo, 2015). Hence, the firing phase of grid and place cells in the hippocampal formation is the only robust correlate of movement direction identified thus far, raising the question of which upstream circuits provide the requisite input.

Although these simulations demonstrate that prominent and sustained rhythmicity is not necessary to support phase coding, they do not address the question of why an arrhythmic state might be preferred in the human brain (Bush & Burgess, 2019). We have shown that it is possible to encode an additional variable in the ongoing frequency of the baseline signal, although this does not necessitate variability over a wide frequency range. Indeed, running speed is encoded in the frequency of rodent hippocampal theta oscillations, which typically vary by <1 Hz (Jeewajee, Barry, et al., 2008a). An alternative possibility is that changes in baseline frequency are used to switch between encoding and retrieval modes of operation, modulating synaptic plasticity by adjusting the temporal interval between firing in connected cells without affecting their relative phase. Indeed, it has been demonstrated that environmental novelty—which might be associated with enhanced learning—reduces theta frequency in the hippocampus (Jeewajee, Lever, Burton, O'Keefe, & Burgess, 2008b) by reducing the slope of the theta frequency versus running speed relationship (Wells et al., 2013). In addition, it has been proposed that encoding and retrieval processes may be segregated by oscillatory phase (Hasselmo, Bodelon, & Wyble, 2002), and this is supported by recent empirical work showing that the preferred theta firing phase of hippocampal place cells also changes during novelty (Douchamps, Jeewajee, Blundell, Burgess, & Lever, 2013).

Phase coding may also offer functional advantages for cognition beyond those considered here. In the context of the rodent hippocampal formation, for example, it has recently been demonstrated that the offline “replay” of behavioral trajectories in place cell activity relies on robust theta sequences during learning (Drieu, Todorova, & Zugaro, 2018). Interestingly, the emergence of robust theta sequences during development also coincides with the disappearance of infantile amnesia (Farooq & Dragoi, 2019; Muessig, Lasek, Varsavsky, Cacucci, & Wills, 2019; Travaglia, Bisaz, Sweet, Blitzer, & Alberini, 2016). These results suggest that the temporal organization of hippocampal spiking activity during active navigation contributes to robust memory encoding. In addition, theta sequences might be useful for the prospective evaluation of upcoming locations or trajectories during active navigation (Bicanski & Burgess, 2018; Bush et al., 2015; Johnson & Redish, 2007). Hence, the phase coding of sensory information during active experience is also likely to contribute to the flexible planning of subsequent behavior.

Finally, although these results demonstrate that it is theoretically possible to maintain a robust phase code in the absence of a baseline oscillation of approximately constant frequency, it is also important to consider how such phase coding might be practically established in real neural circuits. In these simulations, we have assumed that the intrinsic firing frequency of grid cells exceeds that of the baseline oscillation by a value that is proportional to running speed and inversely proportional to grid scale (Figures 2g and 3i). Because phase is the time integral of frequency, and distance the time integral of running speed, this ensures that firing phase encodes displacement through the firing field. Hence, any mechanism that can dynamically maintain the intrinsic firing frequency of a cell above baseline by an amount that is proportional to running speed through the firing field is sufficient to produce robust phase coding. The most parsimonious explanation for this precise frequency difference between baseline and active oscillatory signals being maintained over long periods is that the baseline frequency (assumed here to be represented by the LFP) reflects the average frequency of velocity controlled oscillatory inputs with different preferred firing directions (Burgess, 2008; Burgess, Recce, & O'Keefe, 1993; Geisler et al., 2007, 2010; Hasselmo, 2008; Welday, Shlifer, Bloom, Zhang, & Blair, 2011). Under such a scheme, the LFP signal effectively reflects input from a population of cells whose intrinsic firing frequency varies as a cosine function of movement direction.

Importantly, this proposed mechanism for phase coding need not be restricted to spatial cognition (e.g., Terada, Sakurai, Nakahara, & Fujisawa, 2017)—any variable can be encoded in the phase of neural firing if the difference between intrinsic firing frequency and LFP baseline frequency is proportional to the time derivative of that variable. This raises the possibility that differential frequencies in active neurons and the LFP represent a domain general mechanism in support of phase coding. Crucially, as described above, this frequency difference can be established and dynamically maintained if the LFP signal simply corresponds to the smoothed average population activity (Burgess et al., 1993; Geisler et al., 2007, 2010). This is consistent with the hypothesis that extracellular voltage recordings from a specific region (i.e., the LFP) primarily reflect net synaptic input to that region, if we assume that population activity is a linear function of that input (Buzsaki, Anastassiou, & Koch, 2012). Indeed, in these simulations, filtering the multi‐unit activity of all grid cells in the same 2–20 Hz low frequency band as the LFP signal consistently provides a good substitute for that signal (Figure 7d,e, see Section 2). In sum, we have demonstrated that phase coding in grid cells is not contingent on sustained rhythmicity in neural activity and offers several computational advantages over a pure rate code. Hence, it seems likely that the arrhythmic activity patterns observed in the human brain do not preclude a role for phase coding, and that such a code could make a unique contribution to higher cognitive functions.

## Data Availability

Data available on request from the authors
